# ZIF-8 as Potential Pesticide Adsorbent Medium for Wastewater Treatment: The Case Study of Model Linuron Extraction Conditions Optimization via Design of Experiment

**DOI:** 10.3390/molecules30122480

**Published:** 2025-06-06

**Authors:** Nicola di Nicola, Mariacristina Di Pelino, Martina Foschi, Rosalba Passalacqua, Andrea Lazzarini, Fabrizio Ruggieri

**Affiliations:** 1 Department of Physical and Chemical Sciences (DSFC), University of L’Aquila, Via Vetoio (“A.C. De Meis” Building), 67100 L’Aquila, Italy; nicola.dinicola@graduate.univaq.it (N.d.N.); mariacristina.dipelino@student.univaq.it (M.D.P.); martina.foschi@univaq.it (M.F.); 2 UdR INSTM of L’Aquila, University of L’Aquila, Via Vetoio (“A.C. De Meis” Building), 67100 L’Aquila, Italy; 3 Department of Chemical, Biological, Pharmaceutical and Environmental Sciences & INSTM Lab. CASPE, University of Messina, Viale F. Stagno d’Alcontres 31, 98166 Messina, Italy; rosalba.passalacqua@unime.it

**Keywords:** water remediation, MOFs, DoE, Linuron removal

## Abstract

The increasing presence of pesticide residues in aquatic environments poses a significant threat to ecosystems and human health, necessitating the development of effective removal technologies. In this study, Zeolitic Imidazolate Framework-8 (ZIF-8) was investigated as adsorbent for Linuron, a widely used herbicide. The material was synthesized via a hydrothermal method and underwent thorough physico-chemical characterization, confirming its intrinsic properties. Adsorption experiments were conducted under systematically varied conditions using a Central Composite Face-Centered (CFC) experimental design, evaluating the effects of temperature, Linuron concentration, ionic strength on adsorption efficiency. The Response Surface Methodology (RSM) revealed that temperature and Linuron concentration were the most influential variables. A quadratic effect of ionic strength and a significant interaction between Linuron concentration and ionic strength were also observed. The fitted quadratic regression model exhibited excellent predictive performance (R^2^ = 0.909; Q^2^ = 0.755), and analysis of variance (ANOVA) confirmed its significance (*p* < 0.001) with a non-significant lack of fit. Maximum Linuron removal (>95%) was achieved at elevated temperature, moderate concentration, and intermediate ionic strength. These findings highlight the potential of ZIF-8 as a tunable and high-efficiency adsorbent for the remediation of pesticide-contaminated water, demonstrating the value of RSM-based optimization in designing adsorption processes.

## 1. Introduction

The increasing use of pesticides in agriculture has introduced harmful contaminants into natural water resources, significantly impacting ecosystems and human health. Pesticides are chemical agents that control weeds, insects, fungi, and other pests. While they are intended to be toxic to target organisms, they also pose serious risks to non-target species, including humans. The World Health Organization (WHO) has reported links between pesticide exposure and various chronic health issues, such as neurological disorders, reproductive toxicity, and respiratory diseases [1,2,3]. These chemicals persist in the environment, accumulating in water sources and sediments, and often resist biodegradation due to their stable chemical structures. Given their widespread presence in water systems worldwide, pesticides have become one of the most pressing concerns in water quality management. Linuron, a widely used herbicide, has been identified as an environmental pollutant due to its persistence in aquatic systems and potential toxicity to non-target organisms, including humans [4].

Conventional methods for removing linuron from water, such as chemical degradation and filtration [5,6], often face limitations in efficiency and scalability. Adsorption, on the other hand, has emerged as a promising approach for removing organic pollutants due to its simplicity, cost-effectiveness, and adaptability [7].

Metal–organic frameworks (MOFs) have garnered significant attention in recent years due to their exceptional structural versatility, high surface areas, and tunable physicochemical properties [8,9]. Among these, Zeolitic Imidazolate Framework-8 (ZIF-8), a subclass of MOFs, stands out for its chemical stability, ease of synthesis, and exceptional adsorption capabilities [9,10,11]. ZIF-8, composed of zinc ions coordinated with 2-methylimidazolate linkers, exhibits a sodalite-type topology and a hydrophobic microporous structure [12], making it particularly suitable for applications in adsorption [13], separation, and environmental remediation [12,14].

In this context, ZIF-8 offers unique advantages for linuron adsorption. Its high porosity and hydrophobic nature provide favorable conditions for capturing organic molecules [15], while its stability in aqueous and diverse chemical environments enhances its practicality in real-world applications [16]. Furthermore, the ability to tailor ZIF-8’s properties through functionalization and synthesis optimization opens new pathways for increasing its adsorption efficiency [14].

This study explores the efficacy of ZIF-8 as potential adsorbent for the linuron from either natural or residual water, focusing on optimizing adsorption conditions to maximize removal efficiency. A Design of Experiments (DoE) methodology was implemented to systematically analyze the effects of various parameters on adsorption performance, allowing for the identification of optimal conditions for maximum pesticide removal [17].

## 2. Results and Discussion

### 2.1. Characterization of the Adsorbent Material

**Figure 1:**
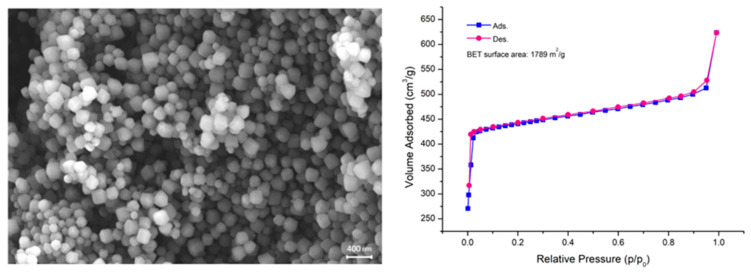
SEM image collected at 20 kX (**left**) and N_2_ adsorption–desorption isotherms at 77 K of ZIF-8 (**right**).

**Figure 2:**
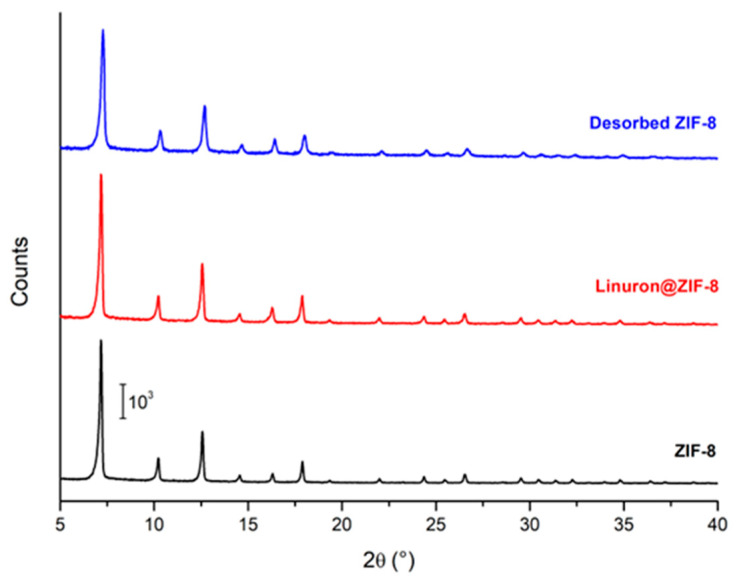
Comparison of the experimental XRPD pattern for pure ZIF-8, Linuron-loaded ZIF-8 and Linuron-desorbed ZIF-8.

ZIF-8 is among the most extensively studied and widely utilized metal-organic frameworks (MOFs), with numerous synthetic strategies available for its preparation [18]. In this study, ZIF-8 was synthesized using an optimized hydrothermal method previously developed by our group [19], therefore we will just give a brief confirmation on the synthetic outcomes. This approach yields ZIF-8 crystals with a uniform truncated−octahedral morphology and an average size of approximately 150 nm, as illustrated in Figure 1. The specific surface area of 1789 m^2^/g [20], determined via nitrogen adsorption–desorption isotherms at 77 K using the BET method, confirmed that the material’s textural properties align with those reported in the literature, further validating the efficiency and reproducibility of the synthesis procedure. Concerning its crystalline structure, from black curve in Figure 2 it is possible to recognize the SOD crystal structure, typical of this class of materials [21].

### 2.2. Response Surface Methodology and Model Fitting

The adsorption capacity of ZIF-8 for linuron was assessed under varying conditions using a Central Composite Design experimental design to systematically explore the impact of temperature, linuron concentration, and ionic strength (salt concentration). Table 1 summarizes the percentage of linuron adsorbed under these experimental conditions. Adsorbed Linuron concentration was obtained by measuring its residual quantity from the treated solutions via HPLC using the calibration described in the Supplementary Materials.

In Table 1 a consistent trend of increased adsorption efficiency is observed at elevated temperatures (e.g., Runs 2, 3, and 5), indicating a strong positive influence of thermal energy on the adsorption process. Conversely, lower removal percentages at 20 °C (e.g., Runs 11, 12, 17) confirm the model’s indication of temperature as a critical factor. The reproducibility of the results is supported by point replicates, which show minimal variation, reflecting low experimental error. These findings validate the robustness of the design and the predictive reliability of the regression model. The significance and contribution of each variable and their interactions in the adsorption process were evaluated through regression analysis. Table 2 presents the quadratic model’s estimated coefficients, standard deviations, confidence intervals, and *p*-values. These results provide insight into each factor’s relative importance and their second-order and interaction effects on the percentage of linuron removal. Terms with statistically significant *p*-values (*p* < 0.05) were considered to have a meaningful impact on the model and were used to interpret the effect of experimental parameters.

**Figure 3:**
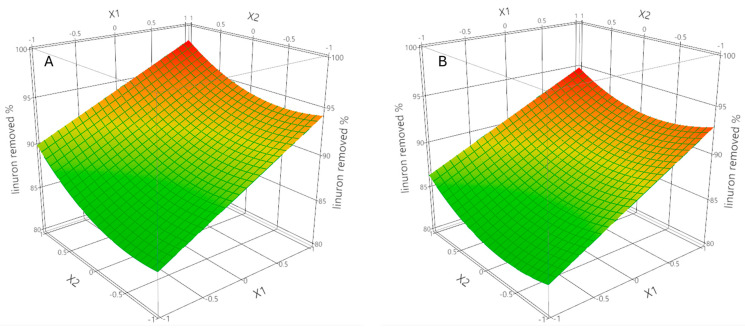
Response surface plots illustrating the interactive effect of temperature (X1 in Table 1) and linuron concentration (X2 in Table 1) on the adsorption efficiency of linuron onto ZIF-8, expressed as percentage removed. In figure (**A**) ionic strength (X_3_ in Table 1) is fixed at the center level and in (**B**) is fixed at the high level. The color scale indicates predicted removal efficiency, where green corresponds to lower values and red indicates higher removal percentages.

The adsorption behavior of Linuron onto ZIF-8 was quantitatively assessed using a second-order polynomial regression model developed from a Central Composite Face-Centered (CFC) design. The model demonstrated excellent predictive and descriptive performance, with R^2^ = 0.909, adjusted R^2^ = 0.873, and a leave-one out cross-validated Q^2^ = 0.755, indicating strong model reliability and generalizability. The lack-of-fit test was non-significant (*p* = 0.5140), confirming that the model adequately fit the experimental data without systematic error. Temperature exhibited the strongest positive linear influence on linuron adsorption as shown in Figure 3. This result is consistent with endothermic adsorption processes, in which elevated thermal energy strongly affects activation barriers for diffusion and improves the interactions between analyte and adsorbent [22,23]. The increased temperature likely promotes higher molecular mobility of linuron in solution and an improved intraparticle diffusion into micro and mesopores of ZIF-8 [24,25]. Another possible effect of temperature is the disruption of weak solvation shells, exposing more active adsorption sites [26,27]. The absence of a significant quadratic term suggests that the positive effect of temperature is linear in the experimental range (20–40 °C), and that no thermal degradation or desorption occurs under the tested conditions [28].

**Figure 4:**
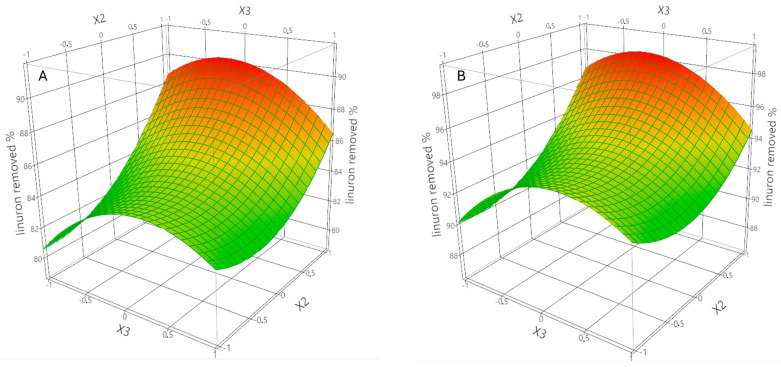
Response surface plots showing the combined effect of Linuron concentration (X2 in Table 1) and ionic strength (X3 in Table 1) on Linuron removal efficiency (%), with temperature (X1 in Table 1) held constant at low level (**A**) and high level (**B**), respectively. The color scale indicates predicted removal efficiency, where green corresponds to lower values and red indicates higher removal percentages.

Linuron concentration had a positive linear effect, indicating that increasing pollutant concentration enhances the driving force for mass transfer [29]. However, the model shows a non-linear adsorption profile with the occurrence of a significant positive quadratic term. Initially, the increase in concentration favors interaction and binding with ZIF-8’s active sites. Nonetheless, at higher levels of concentration, site saturation becomes a limiting factor, and the removal efficiency declines due to the finite number of adsorption sites, as reported in Figure 3. The linear effect of ionic strength was not sufficiently significant, but the quadratic term suggested that the relationship between ionic strength and adsorption efficiency follows a parabolic trend (Figure 4).

Figure 3 shows the response surface plots representing the interaction between temperature and Linuron concentration on the adsorption efficiency. A pronounced increase in Linuron removal is observed with rising temperature, particularly at higher pollutant concentrations [30,31]. This suggests a synergistic interaction where elevated thermal energy enhances molecular mobility and facilitates diffusion into ZIF-8’s microporous structure. The positive curvature associated with concentration reflects initial enhancement of adsorption due to a stronger driving force for mass transfer, followed by a plateau indicative of site saturation at higher levels. These graphical results are fully consistent with the regression model and support the interpretation of an endothermic adsorption mechanism.

Figure 4 illustrates the interaction between Linuron concentration and ionic strength on adsorption efficiency, with temperature held constant at low (A) and high (B) levels. At high ionic strengths, competitive adsorption or ion packing may occur, where Na^+^ or SO_4_^2−^ ions compete with linuron for adsorption sites or modify the hydration shell of the ZIF-8 surface, reducing effective interaction. A medium ionic strength charge reduces repulsion and enhances adsorption by minimizing electrostatic barriers [32]. In the absence of ionic solute, there may be insufficient ionic screening [32,33,34], leading to electrostatic repulsion between Linuron and the ZIF-8 surface. This behavior underscores the dual role of salts in modulating adsorption: acting as both enhancers and inhibitors, depending on concentration [35]. A significant negative interaction between Linuron concentration and ionic strength was observed, suggesting that increasing both simultaneously leads to a decrease in adsorption efficiency; high salt concentrations reduce the solubility of linuron, altering its adsorption behavior [36].

Model predictions indicate that the optimal adsorption conditions for linuron removal onto ZIF-8 occur at 40 °C, 8 μg/mL linuron concentration, and 25 mg/mL Na_2_SO_4_ ionic strength. These conditions provided near-maximal removal efficiencies (≥96%) by leveraging the combined benefits of thermal activation and increased pollutant concentration, while avoiding the adverse effects of excessive ionic content. Notably, the role of ionic strength is crucial in modulating electrostatic interactions between linuron and the ZIF-8 surface and representing the ionic composition of real aquatic environments. At intermediate levels (25 mg/mL), ionic strength reduces electrostatic repulsion, promotes analyte diffusion, and enhances adsorption without triggering competition or surface site shielding that can occur at higher salt concentrations. In addition, this concentration reflects typical ionic strength encountered in surface waters and municipal or agricultural wastewater, thus ensuring that the optimized conditions are environmentally relevant. These findings underscore the importance of considering ionic strength not as a background variable but as an active tuning parameter in the design of adsorption-based water treatment systems. To evaluate the practical applicability of ZIF-8, a preliminary adsorption experiment was conducted using surface water collected from a river near the effluent of a domestic wastewater treatment plant. This matrix, containing natural organic matter and dissolved ions, was spiked with Linuron at 5 μg/mL. The test was performed at 20 °C, under non-optimized yet environmentally relevant conditions. A removal efficiency of 82% ± 2% (mean ± standard deviation, n = 3) was achieved. These preliminary results indicate that ZIF-8 retains substantial adsorption capacity even in complex aqueous environments, supporting its potential use in real water treatment scenarios.

Beyond this validation, a deeper analysis of the experimental results highlights key factors influencing adsorption. Temperature showed the strongest positive effect, consistent with endothermic behavior, where increased molecular motion enhances analyte diffusion and interaction with the adsorbent surface. Linuron concentration exhibited a non-linear trend, suggesting that while higher concentrations initially promote adsorption, site saturation can limit efficiency. The quadratic and interaction terms related to ionic strength indicate a dual role of salts, which can either enhance or hinder adsorption depending on concentration and the presence of competing species. These findings underscore the importance of using multivariate experimental designs to capture complex interdependencies that would be missed with univariate approaches

After optimization and the study of Linuron adsorption, the ZIF-8 used was characterized again to observe the status of possible degradation. The material was characterized immediately after adsorption, with the pesticide molecule inside the pores, and after desorption of the pesticide to study the reversibility of the process. XRPD (Figure 2), ATR-MIR (Figure S1) and SEM (Figure S2) analysis showed no structural, chemical, or morphological differences between the 3 different materials. The XRPD patterns of the loaded and desorbed ZIF-8 show no new peaks and are the same compared to the pattern of pure ZIF-8, indicating that the entire adsorption–desorption process does not alter the crystallinity of the MOF and that the material can be recovered in its pristine form.

## 3. Materials and Methods

### 3.1. Materials

Zinc nitrate hexahydrate (98%), 2-methylimidazole (MeIm, 99%), Na_2_SO_4_ and Methanol (99.8%) were purchased from Sigma-Aldrich (Saint Louis, MO, USA). Linuron standard (purity 99.9%) was obtained from Labor Dr Ehrenstorfer-Schäfers (Augsburg, Germany) and was used without further purification. A stock solution (1.00 g/L) of linuron was prepared by dissolving accurately-weighed 10 mg of pesticide in 10 mL of acetonitrile (HPLC grade, Carlo Erba Reagenti, Milano, Italy) and stored at 4 °C. Deionized water obtained by a water filtration/purification system (Millipore, Bedford, MA, USA) was used.

### 3.2. ZIF-8 Synthesis

Numerous synthetic methods are currently available to produce ZIF-8, even with slight structural variations. For this work, we adapted the synthetic procedure based on the method developed by Koji Kida and coworkers [13]. This method utilizes hydrothermal synthesis, which is particularly well suited for green applications due to the use of water as the solvent, facilitating the production of particles with a high surface area. In brief, 1.488 g of zinc nitrate hexahydrate were dissolved in 40 mL of deionized water and mixed with a solution containing 24.6 g of MeIm in 160 mL of deionized water. The mixture, which rapidly became milky, was stirred at room temperature for 2 h. After the reaction, the resulting suspension was centrifuged at 5000 rpm for 30 min and washed three times with methanol. Finally, the products were dried overnight under reduced pressure at 40 °C.

### 3.3. Adsorption Experiments

For the adsorption experiments, preliminary tests were performed to establish adequate experimental conditions as reported in the Supplementary Materials. Based on these findings, the optimized protocol for further adsorption experiments involved using 10 mg of ZIF-8 added to 7 mL of a 5 μg/mL of linuron solution. The mixture was agitated for 30 min using a magnetic stirrer. Following agitation, the suspension was transferred into a 15 mL Falcon tube and centrifuged at 4000 rpm for 5 min to separate the solid and liquid phases. The supernatants were filtered using 0.22 µm membrane filters to remove the adsorbent and collected for High-Performance Liquid Chromatography (HPLC) analysis to quantify the residual pesticide concentration as reported in the Supplementary Materials. The solid residue (ZIF-8) was washed with 7 mL of acetonitrile (ACN) to extract the adsorbed pesticide, and the extract was also analyzed to confirm adsorption efficiency.

### 3.4. Experimental Design

A Design of Experiments (DoE) approach was employed to optimize the adsorption conditions and identify the most influential factors affecting pesticide removal. The Central Composite Design (CCD), employed with response surface methodology (RSM), was selected due to its ability to evaluate both main effects, interaction effects of the variables and curvatures in multivariate systems. The investigated factors were the temperature (X1), linuron concentration (X2) and ionic strength (X3). Each variable was tested at three levels: low (−1), medium (0), and high (+1) corresponding at the following 20, 30 and 40 °C for X1; 2, 5 and 8 μg/mL for X2 and 0, 25, 50 mg/mL Na_2_SO_4_ for X3. A total of 22 experiments were performed, including replicates at the center point to estimate experimental error. The response variable was the percentage of linuron removed, calculated based on HPLC measurements. Model fitting, and statistical analysis were conducted using Matlab R2020a statistical software. The influence of the three selected factors was modeled by a second-degree polynomial Equation (1).

Y = a_1_ + a_2_·X1 + a_3_·X2 + a_4_·X3 +a_5_·X1·X2 + a_6_·X1·X3 + a_7_·X2·X3 + a_8_·X1^2^ + a_9_·X2^2^ + a_10_·X3^2^ (1)

where Y represents the percentage of linuron removed and a_1_–a_10_ represent the model coefficients that were determined by ordinary least squares regression. In addition, the leave-one-out cross-validation method was used to assess the generalization of the model. The significance of the model and lack-of-fit was estimated by analysis of variance, ANOVA.

### 3.5. Characterization Techniques

#### 3.5.1. XRPD

The XRD patterns of the investigated samples were acquired by a Bruker D2 Phaser benchtop X-ray diffractometer (Bruker AXS GmbH, Karlsruhe, Germany) using Cu Ka radiation operating with the conventional Bragg–Brentano geometry, in the 2θ = 5–40° range. Patterns were acquired with a resolution of 0.05°, acquiring each point for 20 s.

#### 3.5.2. Attenuated Total Reflectance Mid Infrared Spectroscopy (ATR-MIR)

ATR-MIR measurements were carried out using a PerkinElmer (Waltham, MA, USA) Spectrum Two spectrometer equipped with an atmospheric UATR Two accessory and a DTGS detector. Spectra were recorded by averaging 8 scans at a resolution of 4 cm^−1^, covering the spectral range from 4000 to 400 cm^−1^.

#### 3.5.3. Surface Area Analysis

Surface area measurements were conducted using an Anton Paar Nova 800 instrument (Graz, Austria). The sample was placed in a calibrated-volume sample holder and pre-treated at 150 °C under dynamic vacuum for 18 h directly on the instrument. Surface area analysis was performed using N_2_ adsorption–desorption isotherms at 77 K, applying the BET method.

#### 3.5.4. Scanning Electron Microscopy (SEM)

The morphology of the samples was examined via Scanning Electron Microscopy (SEM) using a FESEM ZEISS (Oberkochen, Germany) Gemini500 instrument. Images were acquired at magnifications of 20k, employing backscattered electrons and operating at an accelerating voltage of 5 kV. The samples were mounted on SEM stubs covered with carbon tape and coated with a thin layer of chromium to enhance conductivity and minimize electrical charging during imaging.

## 4. Conclusions

This work presents a comprehensive evaluation of ZIF-8 as an advanced porous material for the removal of Linuron, a persistent and environmentally relevant herbicide, from aqueous media. The MOF was synthesized via a hydrothermal route and thoroughly characterized, confirming the formation of a nanocrystalline and microporous structure with high surface area and chemical stability—key features enabling its adsorption functionality.

The regression analysis revealed that temperature and initial linuron concentration had statistically significant and synergistic effects on removal efficiency, while the influence of ionic strength followed a non-linear trend, with significant quadratic and interaction effects. These findings are consistent with interpretations involving enhanced molecular diffusion, surface accessibility, and competitive interactions modulated by the ionic environment. The resulting second-order polynomial model demonstrated high statistical robustness (R^2^ = 0.909; Q^2^ = 0.755; *p* < 0.001) and predictive accuracy across the design space, with the lack-of-fit test confirming model adequacy. Importantly, a preliminary validation experiment using surface water from a river near a wastewater treatment plant showed a removal efficiency of 82% ± 2% under non-optimized conditions. This result confirms that ZIF-8 maintains substantial adsorption capacity in complex water matrices containing natural organic matter and background ions, thereby reinforcing its potential for real-world application.

This study not only increases the understanding of MOF–pesticide interactions but also highlights the utility of DoE-driven optimization in environmental adsorption science. Future investigations may extend this approach to multicomponent systems, adsorbent regeneration studies, and the assessment of performance in complex water matrices, thus moving toward scalable and sustainable remediation technologies. ZIF-8 was characterized before and after the adsorption process. To investigate the recyclability of MOF after adsorption, the material was washed with MeOH in order to desorb Linuron within the pores of ZIF-8. XRPD, ATR-MIR and SEM analyses performed on the material at each step of the process show that the structural and morphological integrity of the ZIF-8 nanocrystals is not altered, opening up the possibility of reusing the material several times.

## Figures and Tables

**Table 1:** Experimental matrix and results of the CFC design for linuron adsorption onto ZIF-8. The table reports the 22 experimental runs with corresponding values for the three independent variables with their coded levels (X1, X2, X3) and the observed response (percentage of linuron removed). Runs marked with an asterisk (*) are replicates.

Run	Temperature [°C]	Linuron Concentration [μg/mL]	Ionic Strength [mg/mL]	X1	X2	X3	Linuron Removed (%)
1	40	2	0	+1	−1	−1	91.4
2	40	8	50	+1	+1	−1	97.5
3	40	8	50	+1	+1	+1	93.4
4	40	2	0	+1	−1	+1	92.9
5	40	5	25	+1	0	0	92.1
6	30	5	25	0	0	0	91.5
7 *	30	5	25	0	0	0	89.6
8 *	30	5	25	0	0	0	89.1
9	30	2	0	0	−1	0	87.7
10	30	8	50	0	1	0	94.6
11	20	8	50	−1	1	−1	86.9
12	20	5	25	−1	0	0	83.2
13	30	5	25	0	0	−1	85.9
14	30	5	25	0	0	1	88.9
15 *	30	5	25	0	0	−1	85.9
16 *	30	5	25	0	0	1	86.9
17	20	2	0	−1	−1	−1	81.3
18	20	2	0	−1	−1	1	82.7
19 *	20	8	50	−1	1	−1	88.7
20	20	8	50	−1	1	1	85.8
21 *	20	5	25	−1	0	0	86.6
22 *	20	5	25	−1	0	0	86.6

**Table 2:** Summary of the regression model and ANOVA results for linuron adsorption onto ZIF-8. The table includes the estimated significant coefficients (with standard deviations) of the quadratic model terms, along with model performance metrics: coefficient of determination (R^2^), adjusted R^2^, and predictive capability (Q^2^). Terms marked with an asterisk (*) are statistically significant (*p* < 0.05).

Parameters	Value ± SD	R^2^	Adj-R^2^	Q^2^	
intercept	89.5 ± 0.5				
* X1	4.4 ± 0.4				
* X2	2.5 ± 0.4	0.909	0.873	0.755	
X3	0.4 ± 0.4				
* X2·X3	–1.1 ± 0.5				
* X2^2^	1.9 ± 0.7				
**Variation source**	**Sum of squares**	**Degrees of freedom**	**Mean square**	**F-value**	***p*-value**
lack of fit	16.4	8	2.05	0.986	0.5140
pure error	14.5	7	2.1		
model	309.9	6	51.66	25.7	<0.001
residual	30.9	15	2.06		

## Data Availability

Data are contained within the article or Supplementary Materials.

## Supplementary Materials

The following supporting information can be downloaded at: https://www.mdpi.com/article/10.3390/molecules30122480/s1. Preliminary experimental procedure; HPLC calibration procedure; HPLC Analysis; Figure S1: ATR-MIR of ZIF-8 prior and after Linuron discharge; Figure S2: SEM of ZIF-8 prior and after Linuron discharge.
