# Supplementary material for: ZIF-8 as Potential Pesticide Adsorbent Medium for Wastewater Treatment: The Case Study of Model Linuron Extraction Conditions Optimization via Design of Experiment
Source: Molecules. 2025 Jun 6;30(12):2480. doi: 10.3390/molecules30122480 (PMC12195930; doi:10.3390/molecules30122480)
Supplement: Supplementary file 1 [file molecules-30-02480-s001.zip › molecules-3658755-supplementary.pdf]

Supplementary material

ZIF-8 as Potential Pesticide Adsorbent Medium for Wastewater Treatment: The Case Study of Model Linuron Extraction Conditions Optimization via Design of Experiment

Nicola di Nicola [1,2], Mariacristina Di Pelino [1], Martina Foschi [1],
Rosalba Passalacqua [3], Andrea Lazzarini [1,2,*] and Fabrizio Ruggieri [1,*]

1 Department of Physical and Chemical Sciences (DSFC), University of L'Aquila, Via Vetoio ("A.C. De Meis" Building), 67100 L'Aquila, Italy; nicola.dinicola@graduate.univaq.it (N.d.N.); mariacristina.dipelino@student.univaq.it (M.D.P.); martina.foschi@univaq.it (M.F.)
2 UdR INSTM of L'Aquila, University of L'Aquila, Via Vetoio ("A.C. De Meis" Building), 67100 L'Aquila, Italy
3 Department of Chemical, Biological, Pharmaceutical and Environmental Sciences & INSTM Lab. CASPE, University of Messina, Viale F. Stagno d'Alcontres 31, 98166 Messina, Italy; rosalba.passalacqua@unime.it
* Correspondence: andrea.lazzarini@univaq.it (A.L.); fabrizio.ruggieri@univaq.it (F.R.)

Preliminary experimental procedure:

For the adsorption experiments, preliminary tests involved mixing 5 mL of an aqueous pesticide solution (5 µg/mL) with 10 mg of ZIF-8 in a vial, followed by agitation for 1 hour. After mixing, the suspensions were transferred to Falcon tubes and centrifuged at 4000 rpm for 5 minutes. The supernatants were analyzed using High-Performance Liquid Chromatography (HPLC) to determine the residual pesticide concentration. Subsequently, the aqueous phase was removed, and the remaining ZIF-8 solid was washed with acetonitrile (ACN) to desorb and recover the pesticide, providing an estimate of the amount trapped within the framework.

A kinetic study was conducted at three time intervals: 30, 60, and 90 minutes. In this phase, 10 mg of ZIF-8 was introduced into vials containing 7 mL of a 5 µg/mL Linuron solution. The vials were shaken and then transferred into 10–15 mL Falcon tubes and centrifuged for 5 minutes at 4000 rpm. The upper supernatant was collected for analysis, and the residual liquid was removed. The precipitate was then treated with 7 mL of acetonitrile, agitated, and the resulting supernatant was transferred for final analysis.

Results from these tests indicated that the adsorption efficiency remained relatively constant (10–12%) regardless of contact time, and 30 minutes was selected as the standard duration for subsequent experiments. The effect of ionic strength was assessed by preparing a 100 µg/mL calcium chloride solution in phosphate buffer (pH = 6.0) and using it to dilute the Linuron solution. A 10% decrease in pesticide peak area post-adsorption confirmed modest binding under these conditions. Comparable adsorption values (~10%) were observed at pH 7.0 with phosphate buffer.

To enhance removal, the ZIF-8 dose was increased to 50 mg, resulting in an adsorption efficiency of ~90%, confirmed through methanol extraction. Using 25 mg of ZIF-8 slightly reduced adsorption, a trend consistent with the desorption analysis. To evaluate phosphate competition, adsorption was repeated in deionized water, yielding an efficiency of 70.2%.

Calibration Curve

A calibration curve for the quantification of linuron was established in the concentration range of 0.2–10 µg·mL^{-1}. Each concentration level was analyzed in triplicate under identical experimental conditions. A strong linear relationship was observed between analyte concentration and detector response. The resulting regression equation was:

$$y = 92233x + 6574$$

where y represents the chromatographic area and x the concentration of linuron (µg·mL^{-1}). The correlation coefficient was $R^2 = 0.9996$, indicating excellent linearity across the examined range. The standard error of the estimate (SEE) was 4985, confirming the minimal dispersion of the data around the regression line.

The standard error of the slope was 311, and the standard error of the intercept was 1745, supporting the reliability of the regression parameters. Residuals analysis showed no systematic deviation, and precision at each calibration level was satisfactory. In particular, the relative standard deviation for the 0.2 µg·mL^{-1} triplicates was below 1%, highlighting excellent repeatability at low concentration levels.

Sensitivity parameters were calculated following international guidelines. The limit of detection (LOD) was found to be 0.175 µg·mL⁻¹, and the limit of quantification (LOQ) was 0.529 µg·mL⁻¹, using the formulas:

$$LOD = \frac{3.3\,\sigma}{S}; \qquad LOQ = \frac{10\,\sigma}{S}$$

where σ is the standard deviation of the residuals and S is the slope of the calibration curve.

These results confirm that the method is highly sensitive, precise, and well-suited for the quantitative determination of linuron in aqueous samples at trace levels.

HPLC Analysis:

High-performance liquid chromatography (HPLC) was employed to quantify the residual concentration of Linuron in solution. Analyses were performed using a C18 reverse-phase column (250 mm × 4.6 mm, 5 µm particle size) with a mobile phase composed of acetonitrile and water (70:30 v/v). The flow rate was set to 1.0 mL/min, and the injection volume was 20 µL. Detection was carried out using a UV-Vis detector at a wavelength of 248 nm. Each sample was analyzed in triplicate, and calibration curves were constructed from standard solutions of known Linuron concentrations to ensure accurate quantification.

ATR-MIR:

From the ATR-MIR spectra of pure ZIF-8 (black), of ZIF-8 loaded with Linuron (red) and of the same sample then washed with MeOH to remove the pesticide (blue), we can state that the entire adsorption-desorption process does not lead to degradation of the material, in fact all the spectra obtained are perfectly superimposable and no new peaks are observed (Figure S1).

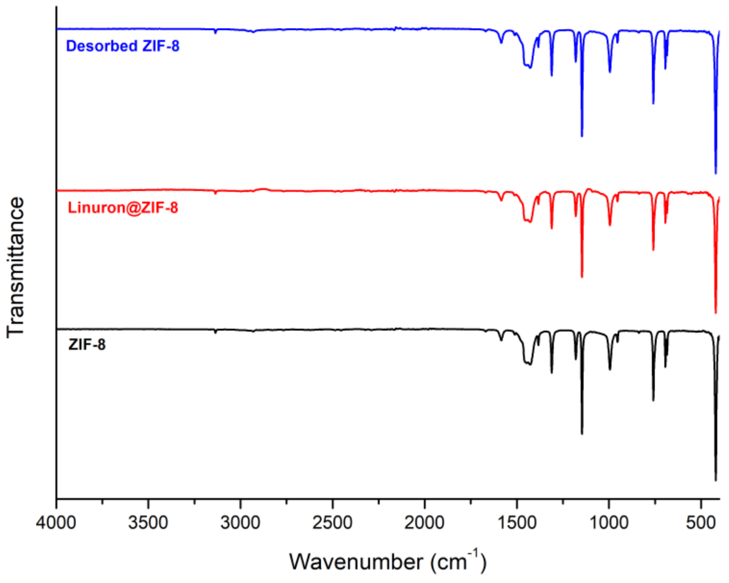

Figure S1: ART-MIR spectra of ZIF-8, Linuron@ZIF-8 and Linuron-desorbed ZIF-8

SEM images show that the adsorption process of Linuron does not lead to any degradation of the ZIF-8 crystals, and it can be seen that the nanoparticle size remains unchanged (Figure S2).

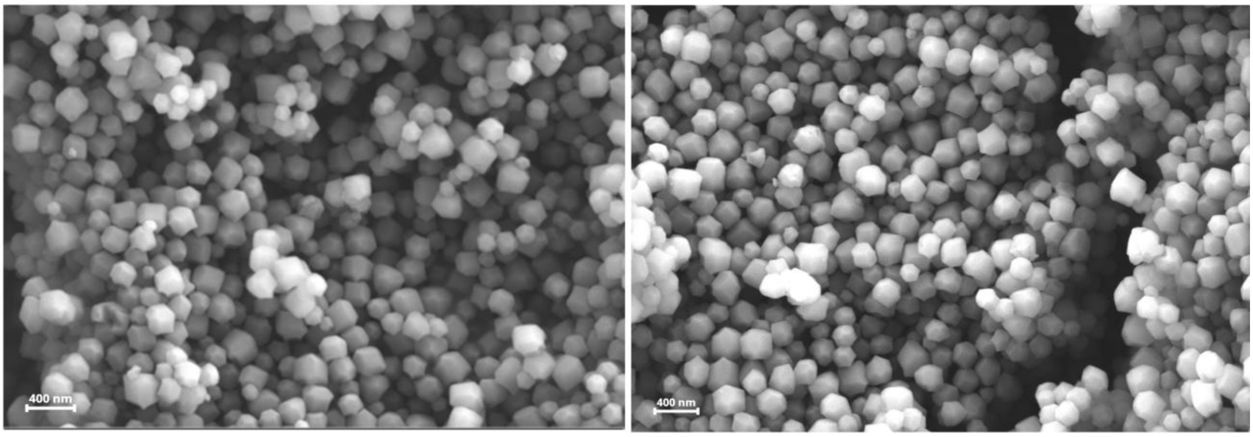

Figure S2: SEM images collected at 20 kX of Linuron@ZIF-8 (left) and Linuron-desorbed ZIF-8 (right).
